# Hemocompatibility and hemodynamic comparison of two centrifugal LVADs: HVAD and HeartMate3

**DOI:** 10.1007/s10237-022-01686-y

**Published:** 2023-01-17

**Authors:** Antonio Gil, Roberto Navarro, Pedro Quintero, Andrea Mares

**Affiliations:** CMT-Motores Térmicos, Universitat Politècnica de València, Camino de Vera, S/N, 46022 Valencia, Spain

**Keywords:** HVAD, HM3, Blade, Gap, Efficiency, Hemolysis

## Abstract

Mechanical circulatory support using ventricular assist devices is a common technique for treating patients suffering from advanced heart failure. The latest generation of devices is characterized by centrifugal turbopumps which employ magnetic levitation bearings to ensure a gap clearance between moving and static parts. Despite the increasing use of these devices as a destination therapy, several long-term complications still exist regarding their hemocompatibility. The blood damage associated with different pump designs has been investigated profoundly in the literature, while the hemodynamic performance has been hardly considered. This work presents a novel comparison between the two main devices of the latest generation–HVAD and HM3–from both perspectives, hemodynamic performance and blood damage. Computational fluid dynamics simulations are performed to model the considered LVADs, and computational results are compared to experimental measurements of pressure head to validate the model. Enhanced performance and hemocompatibility are detected for HM3 owing to its design incorporating more conventional blades and larger gap clearances.

**Supplementary Information:**

The online version contains supplementary material available at 10.1007/s10237-022-01686-y.

## Introduction

Cardiovascular diseases are the leading cause of death globally, entailing around a 30% of all global deaths (World Health Organization 2021). Among them, heart failure (HF) is a disease in which the heart is unable to pump the blood flow required to oxygenate every organ in the body (Ponikowski et al. 2014). For patients with end-stage HF, transplantation is the main treatment option. Nevertheless, due to the increasing number of patients suffering it and the limited compatible donor hearts, Mechanical Circulatory Support (MCS) using Left Ventricular Assist Devices (LVAD) is being increasingly applied as an alternative. Initially, it was used as a short-term bridge to transplantation treatment, while nowadays it is used as a long-term destination therapy (McMurray et al. 2012). However, some complications regarding the hemocompatibility of these devices still exist, such as hemolysis (red blood cells damage) and thrombosis (blood clotting) (Bluestein et al. 2010).

There are different methods to analyze the performance of these devices. Once implanted in a patient, the LVAD-associated blood damage can be determined in vivo (Ochsner et al. 2017) by observing clinical outcomes and blood trauma indicators. Ex vivo (Noor et al. 2016; Petrou et al. 2018; Boës et al. 2019) and in silico (Thamsen et al. 2015; Wiegmann et al. 2019; Zhang et al. 2020) investigations can also be carried out, by testing or modeling the LVAD, respectively. Over the last decades, the use of computational fluid dynamics (CFD) has been extended to the evaluation of hemodynamics of biomedical devices due to its numerous advantages (Shi and Korakianitis 2018). Simulations can provide data on regions that have a difficult access for measuring or visualizing, and predict physical quantities which are difficult to obtain experimentally, allowing the evaluation of the device without the need of producing costly prototypes (Malinauskas et al. 2017). However, an experimental validation of the model is required to prove the reliability of numerical results. Moreover, the USA Food and Drug Administration (FDA) developed a benchmark centrifugal blood pump to be tested and modeled by multiple research groups in order to use it as a validation tool and standardize the use of CFD in the investigation of blood pumps. Both experimental (Hariharan et al. 2018) and computational (Good and Manning 2020; Karimi et al. 2021) studies of the FDA blood pump have been carried out to evaluate CFD models and validate them using measurement data collected by different laboratories.

LVADs based on continuous-flow turbopumps have replaced earlier pulsatile-flow devices based on volume displacement pumps, owing to their smaller size and improved durability (Timms 2011). Although continuous-flow devices can cause some long-term complications due to the lack of pulsatility, their simpler designs involve fewer moving parts leading to a more reliable option (Fraser et al. 2012). Turbopumps, for its part, are divided into axial and centrifugal pumps. Generally, axial pumps require faster rotational speeds to operate, involving higher mechanical loses and increased shear stresses. Fraser et al. (2012) compared several devices of both types and detected higher mean and maximum shear stresses and larger percentage of volumes exposed to high levels of shear stress in axial pumps, as well as longer exposure times to elevated shear stresses. This results in higher levels of hemolysis in axial pumps compared to centrifugal ones. Moreover, in axial pumps the impeller suspension is achieved by means of a mechanical bearing which incorporates contacting surfaces that are potential sites for thrombus deposition (Moazami et al. 2013). In contrast, the geometry of the impeller in centrifugal pumps allows for a contactless bearing system using hydrodynamic or magnetic levitation (Timms 2011). However, an inappropriate design of the centrifugal pump can lead to significant levels of blood damage, even greater than damage levels found in axial pumps. Thamsen et al. (2015) compared the blood damage of two devices, HeartMate2 (axial) and HeartWare VAD (centrifugal), and obtained similar levels of hemolysis. In fact, the centrifugal pump presented slightly larger volumes exposed to elevated shear stresses as well as higher residence time of blood within the pump, both contributing to hemolysis. They concluded that the design of HeartWare VAD was generating a level of hemolysis comparable to that found in the axial pump. This design involved extremely narrow gaps and wide areas between impeller blades and housing, and the highest damage was detected in the gap region. Zhang et al. (2020) compared those pumps with CH-VAD, a newer maglev centrifugal pump that was in preclinical evaluation, and detected similar levels of blood damage for HeartWare VAD and HeartMate2, while the predicted level of hemolysis was two times lower for CH-VAD. The enhanced hemocompatibility of this pump was attributed to its flow path design which led to reduced flow recirculation. However, in these works they did not perform a parallel analysis about the hemodynamic performance of the pumps, which could be related with their hemocompatibility. Gil et al. (2022) conducted a detailed analysis on the performance of HeartWare VAD to justify the causes of its elevated risk of hemolysis. They concluded that the extremely low gap clearances and the large blade tip areas in contact with the gap region were inducing a high shear stress level in this region, and that large zones of flow recirculation within the blade-to-blade passages were worsening the pump performance as well. These phenomena led to significant levels of hemolysis as well as reduced efficiency.

The latest generation of devices is composed of centrifugal pumps with magnetic levitation bearings. HeartWare VAD (HVAD) employs a hybrid levitation system to position the impeller through the balance of magnetic and hydrodynamic forces. Due to the hydrodynamic lift requirements, it has a 4-wide-blade impeller whose large top and bottom surfaces are tapered, and axial clearance gaps between casing and impeller are extremely narrow, of the order of ten blood cells. Passive magnetic bearing, for its part, is generated between center post’s coils and impeller’s magnets (Foster 2018). HeartMate3 (HM3) achieves the levitation and rotation of the impeller using a fully magnetic system, through a combination of passive and active magnetic forces produced between casing’s coils and impeller’s permanent magnets (Foster 2018). The active magnetic levitation system allows the operation with no liquid working fluid. In contrast to HVAD, clearance gaps in HM3 are relatively large, of the order of hundreds of blood cells. Hybrid and fully magnetic levitation systems have completely replaced mechanical bearings to avoid friction, heating and dynamic sealing, which reduces blood damage risk (Wu et al. 2021). Nonetheless, HVAD was recently withdrawn from the market owing to elevated thrombogenicity. However, the analysis of this device is still important, due to the existence of patients which were implanted with HVAD before its removal.

In the current work, these two devices are analyzed using CFD, and computational models are validated through ex vivo experimental tests. The main objective of this work is to compare the performance of both devices. To the authors’ knowledge, this is the first time HVAD and HM3 are compared using CFD in terms of both hemodynamics and hemocompatibility. Boës et al. (2019) investigated these devices experimentally together with two axial pumps, but they focused on the construction of a universal 0D model based on principles of turbomachinery for rotary blood pumps. Boraschi et al. (2021) also evaluated these devices using CFD, but they focused on their hemocompatibility during the artificial pulse operating condition.

The document is structured as follows. Firstly, the methodology is described including both the experimental test bench configuration and the computational set-up of the simulations. The hemolysis model implemented in the simulations is explained in detail as well. Secondly, the results are presented. The pressure head of both devices working at several operating conditions is measured and compared to CFD predictions. Then, the considered devices are compared using the operating maps of the pumps, the volumetric distribution of shear stresses and several fluid patterns. Moreover, the devices are compared from both perspectives: the hemodynamic performance and the associated blood damage. Next, the results are discussed. Finally, the conclusions derived from the results are exposed, highlighting the contributions of the work, and the limitations of the study are declared as well. Additionally, the results of the mesh independence study and the comparison between steady- and unsteady-state results are presented as Supplementary Material.

## Materials and methods

### Experimental set-up

**Fig. 1:**
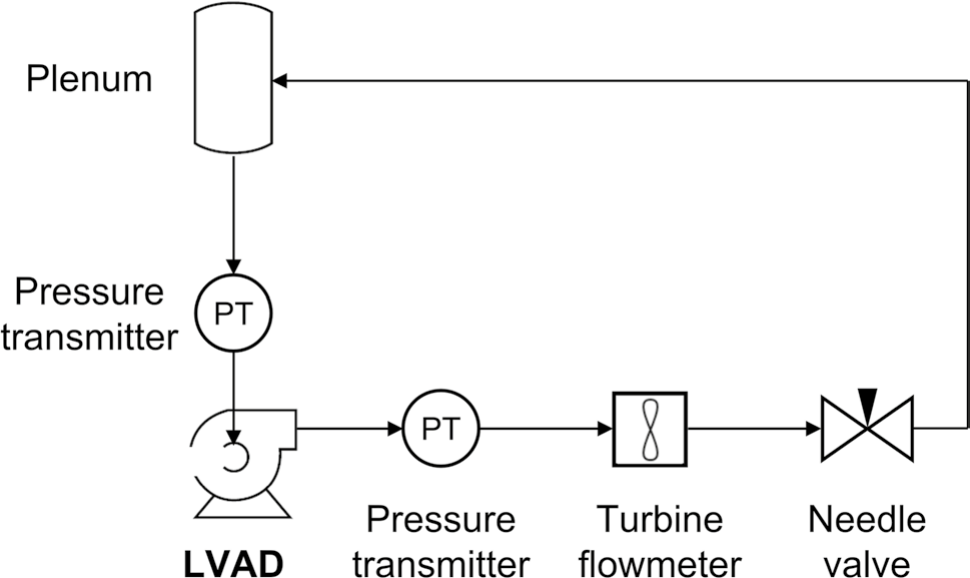
Sketch of the experimental tests flow loop

The pressure head maps of both devices are obtained experimentally to validate the CFD models. The devices are tested in a closed loop flow circuit operating under continuous-flow conditions at different rotational speeds. The pressure head is measured using two pressure transmitters (WIKA PE 81.61 S-20, [0, 2.5] bar, accuracy of ± 0.006 bar, WIKA Instruments, Germany), allocated at 5 cm from the inlet and outlet of the device. The flow rate through the pump is adjusted using a needle valve. A radial flow turbine flowmeter (RS PRO 257–133, [1.5, 30] L/min, RS Components, UK) is connected downstream of the outlet, at around 15 cm, to measure the flow rate with a resolution of 5×10^–4^ L/min. A distilled water–glycerol mixture (40% glycerin) is used to simulate the blood viscosity. The density and viscosity of this working fluid are $\rho = 1100{\text{ kg}}/{\text{m}}^{3}$ and $\mu = 3.5{\text{ mPa}} \cdot {\text{s}}$, respectively. A sketch of the flow loop is shown in Fig. 1, representing the experimental test bench configuration.

### Computational set-up

Computational fluid dynamics simulations are performed to evaluate the flow within the considered LVADs. The CFD software employed in this work is Simcenter STAR-CCM + (Siemens). The blood is modeled as a liquid with density $\rho = 1060 {\text{kg}}/{\text{m}}^{3}$. It is assumed to be a Newtonian fluid, i.e., with constant viscosity $\mu = 3.5 {\text{mPa}}\cdot{\text{s}}$, since its non-Newtonian behavior is expected to be negligible where shear rates are greater than $100 {\text{s}}^{ - 1}$, such as those found in VADs (Fraser et al. 2011; Wang et al. 2019; Wiegmann et al. 2019). However, the non-Newtonian behavior of blood may be manifested in regions with lower shear rates. Therefore, additional simulations were performed to prove the applicability of this assumption, and small discrepancies were found.

Given a working fluid, the fluid dynamics features within the device are entirely defined by the rotational speed of the impeller ($\Omega$) and the volumetric flow rate through the pump ($Q$). The operating map consists in the representation of the performance variables against the volumetric flow rate for several values of rotational speed. The performance variables of interest when investigating a hydraulic pump are pressure head (${\Delta }p_{t}$), mechanical shaft power ($P$) and efficiency ($\eta$) defined as the ratio between the hydraulic energy transferred to the fluid through the pump and the mechanical shaft power supplied to rotate the impeller (Eq. 1).

1 $$\eta = \frac{{Q\Delta p_{t} }}{P}$$

Owing to the incompressible behavior of the working fluid, the flow field through the device is not affected by the mean pressure. Therefore, the reference (atmospheric) stagnation pressure can be imposed elsewhere. In these simulations, this pressure is imposed at the inlet while a mass flow rate equal to $\dot{m} = \rho Q$ is imposed at the outlet.

The fluid field within the pumps is solved in steady-state simulations. The impeller motion is imposed using a Moving Reference Frame (MRF) approach. Therefore, the set of equations of motion for the rotating region are formulated in a reference frame that rotates at the rotational speed of the impeller, i.e., a term of inertial body forces is incorporated to the momentum equation in the rotating region (Torregrosa et al. 2019). In order to avoid circumferential heterogeneity due to the frozen impeller, this approach is applied together with a mixing plane interface between static and rotating regions (Galindo et al. 2020). In the previous work by Gil et al. (2022), the steady MRF approach was compared to the transient sliding mesh approach and, despite the non-negligible discrepancies found for HVAD operating at extreme off-design flow conditions, the steady approach was found to provide fair results compared with experiments at design conditions. HM3, for its part, presents negligible discrepancies between both approaches. The comparison of steady and transient approaches for both devices can be found in the Supplementary Material section. Moreover, the characterization of turbopumps is commonly performed under the assumption of quasi-stationary flow in order to obtain the entire operating map (Jiao et al. 2009). Hence, the stationary methodology is applied for this work owing to the significant reduction of computational cost achieved using this approach, at the expense of neglecting transient effects. The steady-state simulations were performed on an Intel® Xeon® Gold 6248R CPU using 48 parallel processes, and computational times were around 6 s per iteration resulting in 2 to 7 h of calculation.

Reynolds-Averaged Navier–Stokes (RANS) equations are solved to obtain the mean flow solution. These equations are derived from the complete set of mass, momentum and energy conservation equations by imposing the Reynolds decomposition (Pope 2001). The k-ω model with shear stress transport (SST) proposed by Menter (1993) is selected for the estimation of Reynolds stresses to achieve closure of RANS equations. This model applies the standard k-ω model near the wall and the k-ε model in the far-field (Menter 1993). Most authors use the k-ω SST model when simulating blood pumps (Fraser et al. 2012; Gross-Hardt et al. 2019; Thamsen et al. 2020).

**Fig. 2:**
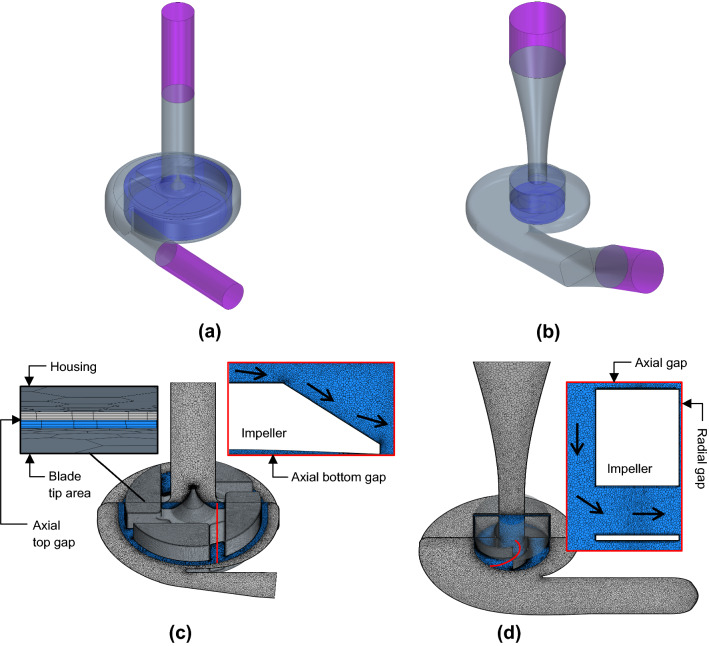
Fluid domain of **a** HVAD and **b** HM3: static region (gray), rotating region (blue) and inlet/outlet cannulas (violet, not to scale). Computational mesh (3D view and 2D meridional view) of **c** HVAD, with a zoom into the gap thin mesh, and **d** HM3

Concerning the computational domain, the CAD models of the devices were obtained by reverse engineering. The CAD of HM3 was created based on measurements and observations of the physical device and geometric data available from the literature. The CAD of HVAD was obtained by 3D-scanning using a HDI Advance 3D scanner which employs structured light technology and delivers high-resolution digital scans with an accuracy of 50 μm, and afterward it was subjected to a subsequent cleanup process to smooth the scanned surfaces. The main dimensions of both devices are listed in Table 1. Figure 2a, b presents the fluid domain of both devices, differencing between static and rotating regions and showing the inlet and outlet cannulas that allocate the boundary conditions sufficiently far from the domain of interest. This domain including long inlet and outlet ducts is not representative of the LVAD inserted in the heart apex, but similar to the experimental test bench configuration. Additional simulations of a more realistic scenario including an ill ventricle upstream of the device were performed, and negligible discrepancies were found. Therefore, the physic phenomena occurring within the pump are assumed to be essentially the same in both configurations.

**Table 1:** Main dimensions of the HVAD and HM3 devices

Parameter	Symbol	HVAD	HM3	Units
Impeller diameter	$D_{{{\text{imp}}}}$	$34.6$	$18.7$	$\mathrm{mm}$
Inlet diameter	$D_{{{\text{inlet}}}}$	$12.75$	$19.7$	$\mathrm{mm}$
Outlet diameter	$D_{{{\text{outlet}}}}$	$10$	$15.2$	${\text{mm}}$
Volute area/radius ratio	$A/R$	$2.26 \times 10^{{ - 3}}$	$3.73 \times 10^{{ - 3}}$	${\text{m}}$
Diffusor radius ratio	$R_{{{\text{in}}}}^{{{\text{dif}}}} /R_{{{\text{out}}}}^{{{\text{dif}}}}$	$0.84$	$0.66$	–
Axial (top) gap clearance	$c_{{{\text{ax}}}}$	$40$	$1000$	μm
Radial gap clearance	$c_{{{\text{rad}}}}$	–	$500$	$\mathrm{\mu m}$

The fluid volume is discretized by means of a polyhedral grid. A mesh independence study is performed for each device. The results of these mesh studies are presented in the Supplementary Material section. As a result of the mesh independence studies, a global mesh size of $8 \times 10^{ - 4} {\text{m}}$ is selected, with refinement sizes of $1 \times 10^{ - 4} {\text{m}}$ in the rotating region and including a 10-element prism boundary layer along walls. A good quality of the boundary layer and a correct resolution of the viscous sublayer are checked through the wall $y^{ + }$, which is less than 1 in most part of the walls: 97.8% of the walls for HVAD and 97.2% for HM3, both operating at the most unfavorable condition (maximum values of rotational speed and flow rate). The resulting meshes are represented in Fig. 2(c,d).

**Fig. 3:**
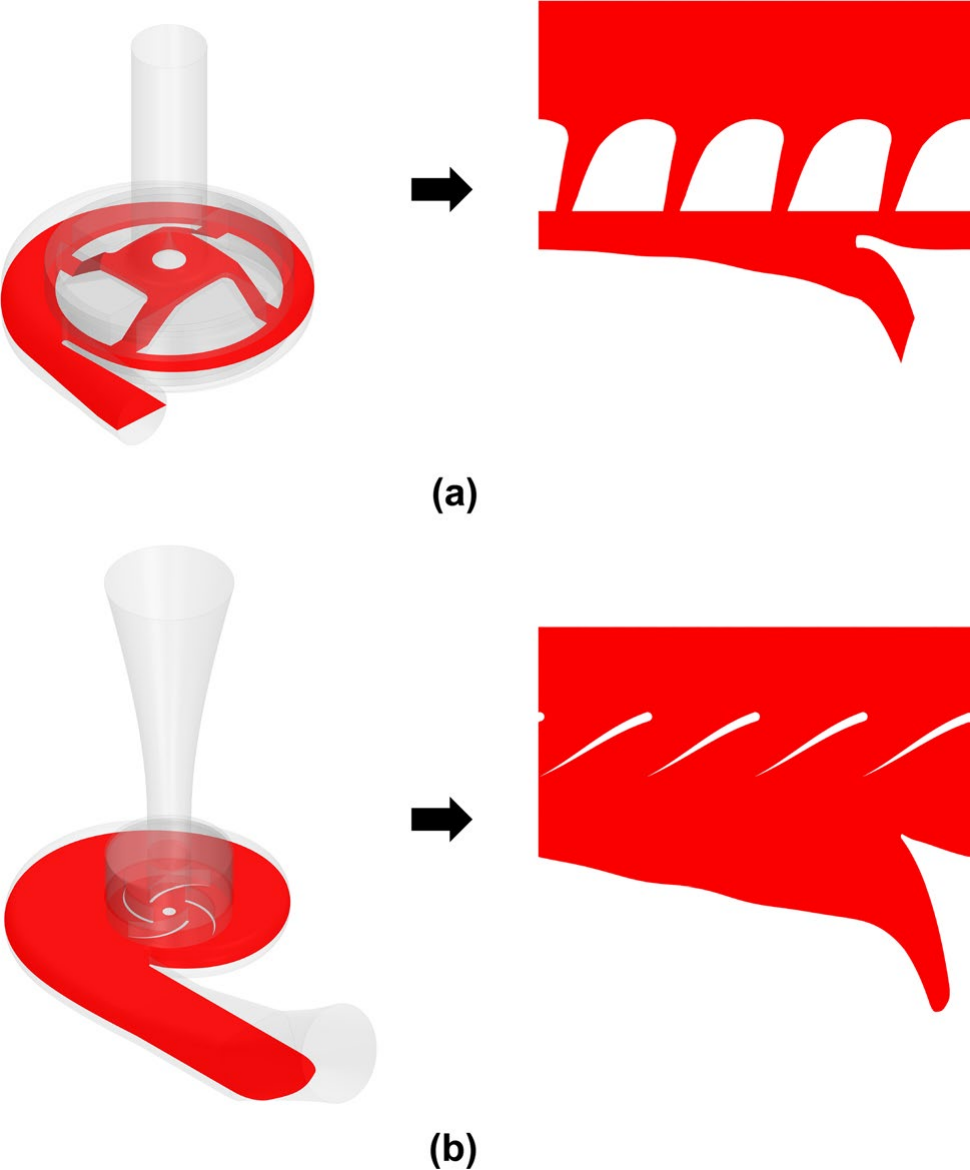
Surface unwrapped for the blade-to-blade representation of the fluid field within **a** HVAD and **b** HM3

To visualize the fluid field within turbopumps, a common practice is to represent it in the midplane of the blade-to-blade passage. Figure 3 shows the represented surface in red color on the left and the resulting blade-to-blade plane on the right.

### Blood damage: hemolysis index model

Hemolysis is the disintegration of red blood cells resulting in a release of hemoglobin into the blood plasma. It is associated to the flow-induced mechanical damage exerted over red blood cells, and is directly related with the scalar shear stress ($\tau$), calculated using Eq. 2

2 $$\tau = \sqrt {\frac{1}{6}\mathop \sum \limits_{i \ne j} \left( {\sigma_{ii} - \sigma_{jj} } \right)^{2} + \mathop \sum \limits_{i \ne j} \sigma_{ij}^{2} }$$

where $\sigma_{ij}$ is the components of the viscous stress tensor (Taskin et al. 2012). It must be noted that the scalar shear stress depends on both shear ($\sigma_{ij}$) and normal ($\sigma_{ii} )$ components of stress. The Reynolds stress components are not considered since Reynolds stress tensor is a statistical quantification of the averaged transport of fluctuating momentum, and has no direct link to physical forces acting over blood cells (Ge et al. 2008).

The power law model presented in Eq. 3 shows the nonlinear dependency of hemolysis index (${\text{HI}}$) on both shear stress and exposure time (Taskin et al. 2012). Here, $\Delta {\text{hb}}$ is the released hemoglobin due to the rupture of blood cells, while ${\text{HB}}$ refers to the total hemoglobin concentration, which normally takes a value of ${\text{HB}} = 10{\text{ g/dL}}$.

3 $${\text{HI}} = \frac{{\Delta {\text{hb}}}}{{{\text{HB}}}} = C \cdot t^{\alpha } \cdot \tau^{\beta }$$

An additional scalar transport equation must be solved to quantify ${\text{HI}}$, since the nonlinearity in time of Eq. 3 prevents its direct application and the calculation of ${\text{HI}}$ at the outlet of the device as the sum of local values based on shear stresses and residence time within each grid cell (Wu et al. 2021). Garon and Farinas (2004) demonstrated the formulation of the transport equation for ${\text{HI}}$. They linearized Eq. 3 in time by applying the variable change $\Delta {\text{hb}}^{\prime } = \Delta {\text{hb}}^{1/\alpha } = \left( {{\text{HB}} \cdot C \cdot \tau^{\beta } } \right)^{1/\alpha } \cdot t$. The resulting transport equation for $\Delta {\text{hb}}^{\prime }$ is presented in Eq. 4, where the diffusion term is excluded.

4 $$\frac{{\partial \left( {\Delta {\text{hb}}^{^{\prime}} } \right)}}{\partial t} + u_{j} \frac{{\partial \left( {\Delta {\text{hb}}^{^{\prime}} } \right)}}{{\partial x_{j} }} = \left( {{\text{HB}} \cdot C \cdot \tau^{\beta } } \right)^{{\frac{1}{\alpha }}}$$

Different values are used in the literature for the empirical constants of the source term on the right-hand side of Eq. 4. In this work, these values are set to be $C = 3.62 \times 10^{ - 7} {\text{s}}^{ - \alpha } {\text{Pa}}^{ - \beta }$, $\alpha = 0.785$ and $\beta = 2.416$, obtained experimentally by Giersiepen et al. (1990) for human blood.

The resolution of a scalar transport equation allows for the Eulerian evaluation of ${\text{HI}}$ in the whole domain. This transport equation is solved imposing ${\text{HI}} = 0$ at the inlet, and the device ${\text{HI}}$ is evaluated as the mass-flow average of this parameter at the outlet, as defined in Eq. 5 (Craven et al. 2019).

5 $${\text{HI}}_{\text{device}} = \frac{{\mathop \smallint \nolimits_{\text{outlet}} {\text{HI}} \left| {{\mathbf{u}}\cdot{\text{d} \! \mathbf{A}}} \right|}}{{\mathop \smallint \nolimits_{\text{outlet}} \left| {{\mathbf{u}}\cdot{\text{d} \mathbf{A}}} \right|}}$$

Taskin et al. (2012) evaluated different procedures for the ${\text{HI}}$ calculation and indicated that available methods are useful in predicting relative hemolysis to compare between several devices or operating conditions, but they do not predict an accurate absolute value of ${\text{HI}}$. Therefore, the relative hemolysis is evaluated by means of the relative hemolysis index (${\text{RHI}}$), calculated as in Eq. 6. The reference value of ${\text{HI}}$ is taken to be that obtained for HM3 operating at $\Omega = 6000\;{\text{rpm}}$ and $Q = 5 L/\min$.

6 $${\text{RHI}} = \frac{{{\text{HI}}}}{{{\text{HI}}_{{{\text{ref}}}} }}$$

## Results

### Experimental validation of the CFD model

**Fig. 4:**
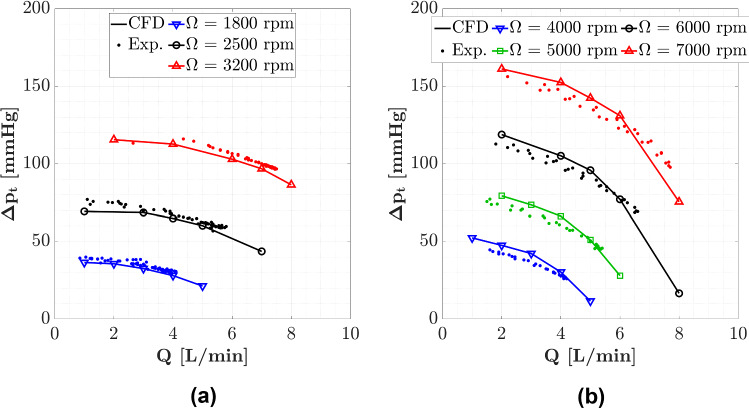
Pressure head against volumetric flow rate for several values of rotational speed, obtained experimentally and numerically for **a** HVAD and **b** HM3

Figure 4 shows the pressure head maps obtained experimentally and numerically. A good agreement is found between experimental measurements and CFD predictions. At nominal conditions, a maximum relative error lower than 5% of the predicted value is detected for both devices.

### Hemodynamic performance

In this section, a comparison between both devices is performed based on their operating maps, and fluid patterns are presented to better understand the physics phenomena occurring within the pumps.

**Fig. 5:**
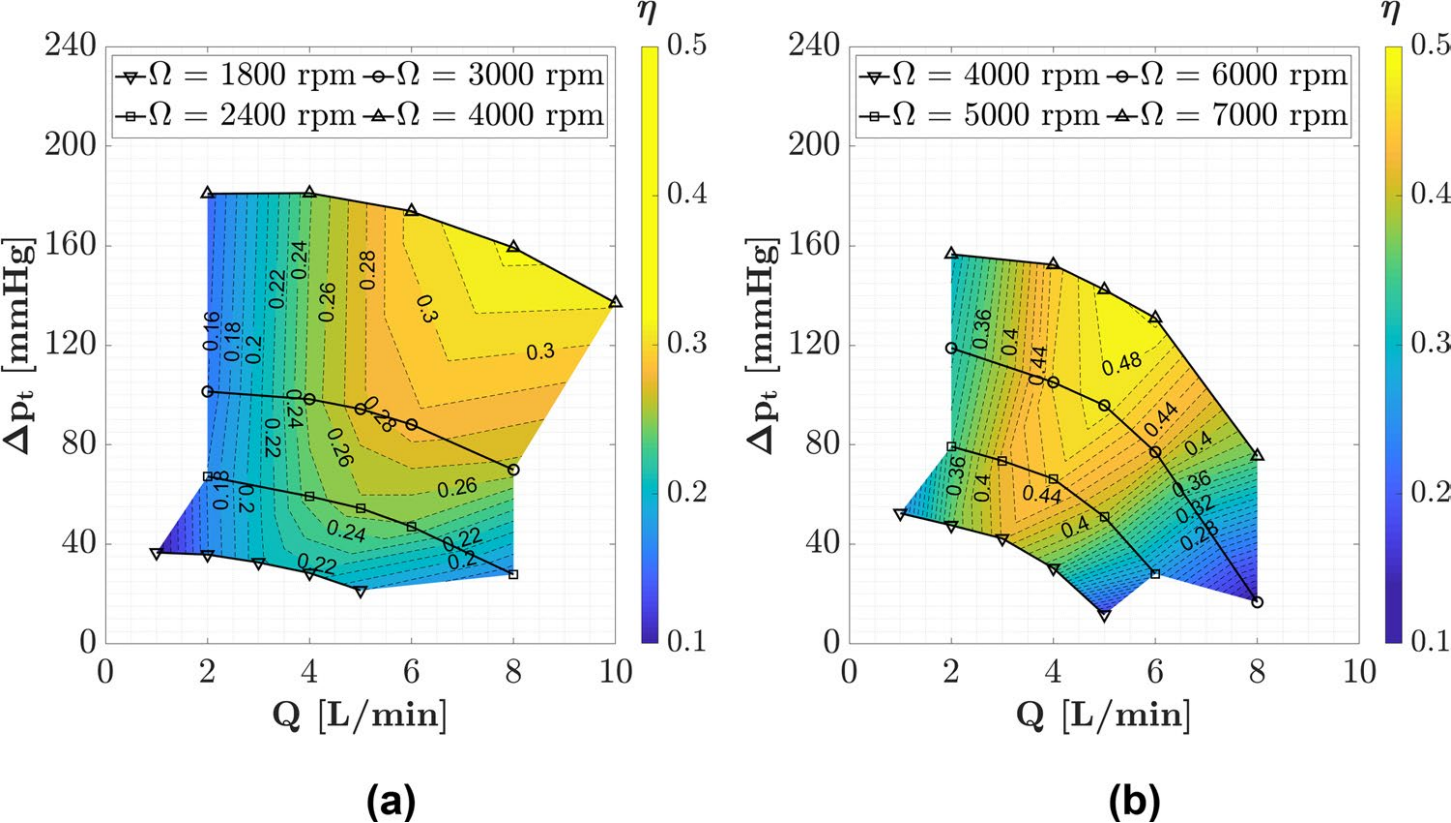
Operating maps of **a** HVAD and **b** HM3, showing pressure head curves against volumetric flow rate and colored contours of efficiency for several values of rotational speed

The operating maps presented in Fig. 5 show the pressure head curves for several values of rotational speed, together with colored contours of hydraulic efficiency.

The nominal rotational speed for each device can be deduced based on previous operating maps. The nominal operating condition must correspond with normal values of cardiac output (CO) and mean arterial pressure (MAP). For a healthy adult weighing around 70 kg, these quantities are ${\text{CO}} \approx 5 {\text{L}}/{\text{min}}$ and ${\text{MAP}} \approx 100\;{\text{mmHg}}$ (Guyton 1983). The desired volumetric flow rate is imposed by the cardiac output: $Q = {\text{CO}}$. The pressure head is equal to the difference between afterload (aortic pressure) and preload (left ventricular pressure). In healthy cardiovascular systems both pressures have a pulsatile profile, taking values between diastole and systole in the following ranges: $p_{{{\text{Ao}}}} \in \left[ {80, 120} \right] {\text{mmHg}}$ and $p_{{{\text{LV}}}} \in \left[ {10,120} \right]\;{\text{mmHg}}$. Assuming that in patients suffering from advanced HF both aortic and left ventricular pressures are almost constant since the failing heart is hardly pumping, $p_{{{\text{Ao}}}} \cong 100\;{\text{mmHg}}$ and $p_{{{\text{LV}}}} \cong 14\;{\text{mmHg}}$ (left ventricular end-diastolic pressure in patients suffering from HF) (Jain et al. 2019). Hence, the nominal value of pressure head required for the LVADs can be expressed as $\Delta p = p_{{{\text{Ao}}}} - p_{{{\text{LV}}}} \cong 86 {\text{mmHg}}$. Therefore, based on Fig. 5, the appropriate values of $Q = 5 {\text{L}}/{\text{min}}$ and $\Delta p_{t} \cong 86 {\text{mmHg}}$ determine nominal values of rotational speed in the range of $\Omega \in \left[ {2400,3000} \right]\;{\text{rpm}}$ for HVAD and $\Omega \in \left[ {5500, 6000} \right] {\text{rpm}}$ for HM3. From now on, nominal rotational speed will refer to $\Omega_{{{\text{nominal}}}}^{{{\text{HVAD}}}} = 3000{\text{ rpm}}$ and $\Omega_{{{\text{nominal}}}}^{{{\text{HM3}}}} = 6000{\text{ rpm}}$ for HVAD and HM3, respectively.

**Fig. 6:**
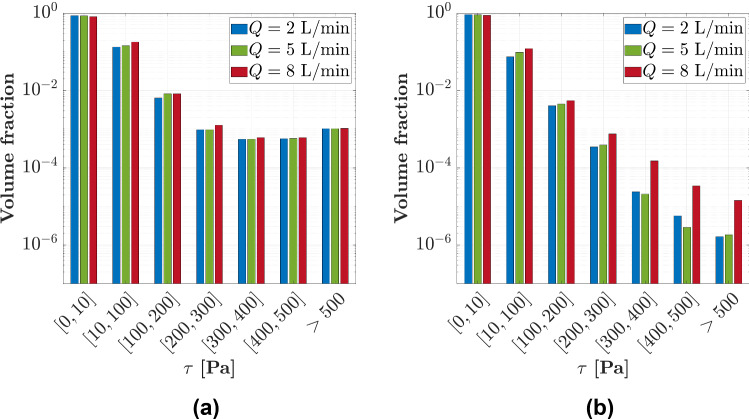
Volumetric distribution of the scalar shear stress within **a** HVAD and **b** HM3, operating at their nominal rotational speeds and for nominal and extreme values of flow rate

The volumetric distribution of scalar shear stresses within each device operating at nominal rotational speed is presented in Fig. 6, showing its dependency with flow rate.

**Fig. 7:**
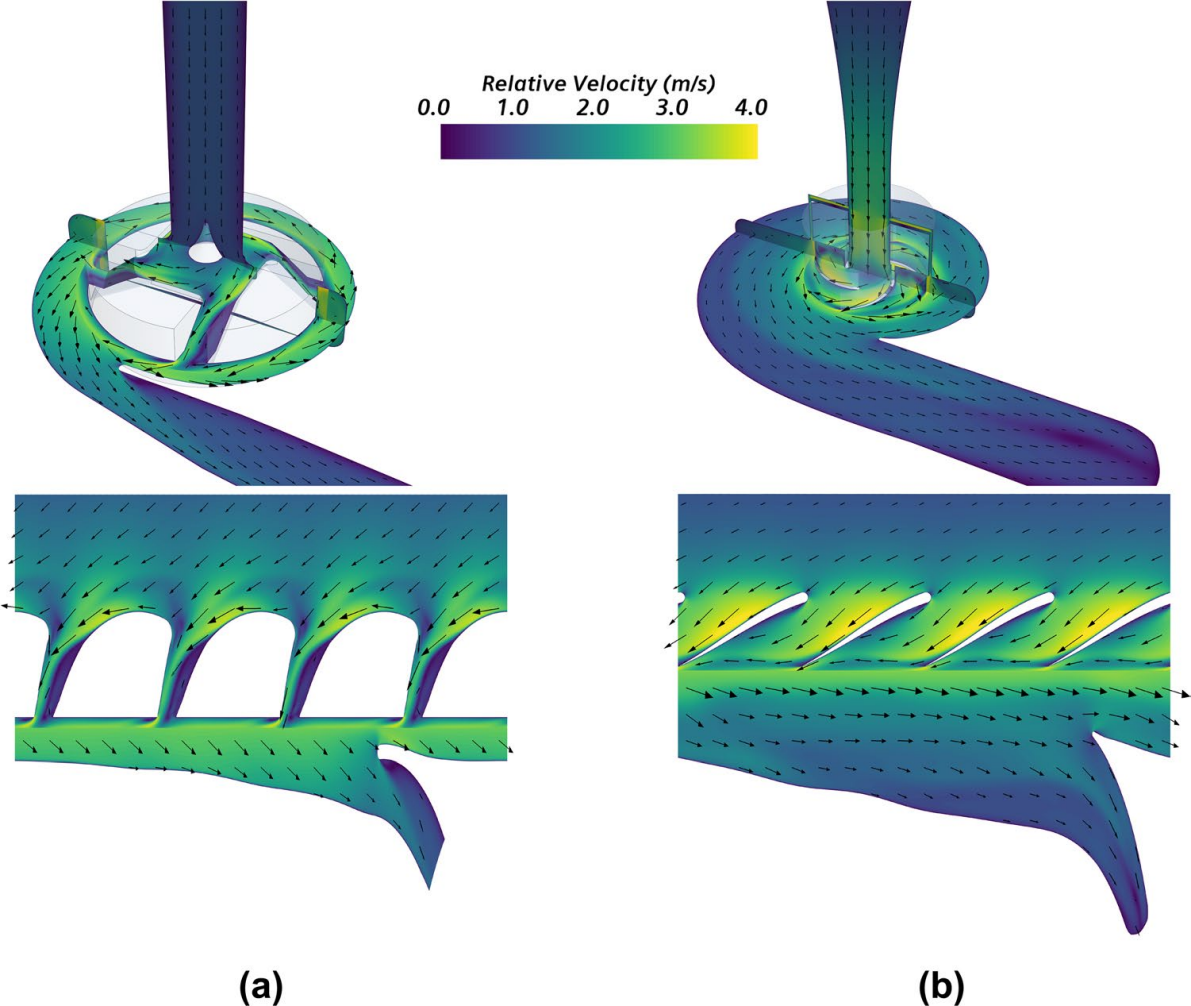
3D view (up) and blade-to-blade representation (down) of the relative velocity field within **a** HVAD and **b** HM3, operating at nominal conditions (*Q* = 5 L/min, *∆p*_*t*_ ≅ 90 mmHg)

**Fig. 8:**
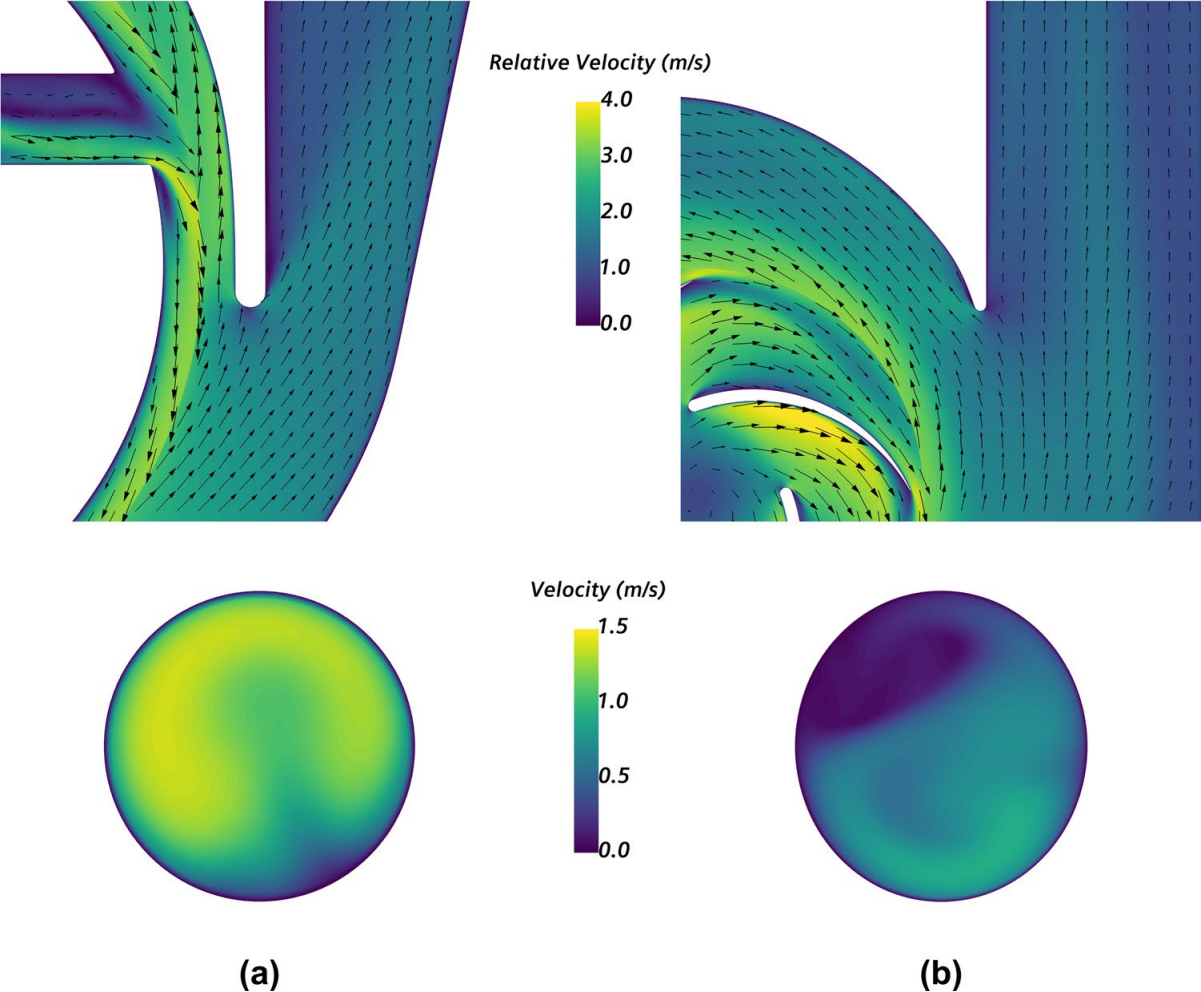
Velocity field round the volute tongue (up) and at the outlet cross section (down) for **a** HVAD and **b** HM3, operating at nominal conditions (*Q* = 5 L/min, *Δpt* ≅ 90 mmHg)

Fluid patterns within each device are presented to better understand the hemodynamic performance of HVAD and HM3. Figure 7 shows the blade-to-blade representation—obtained as shown in Fig. 3 and a 3D view of the relative velocity field within both devices operating at nominal conditions. Figure 8 presents the velocity field round the volute tongue together with the velocity pattern at the outlet cross section for each device. The velocity field within each region must be represented in an appropriate reference frame. The relative velocity in a static region equals the absolute velocity since it is referred to static walls. Contrarily, the relative velocity in a rotating region is referred to the moving walls in order to detect zones of recirculating flow. Thus, the relative velocity in the rotating region is represented in the MRF, and the discontinuity in the velocity field detected at the interface between rotating and static regions is due to the change from moving to stationary reference frames (Karimi et al. 2021). It must be noted that the colormap in Fig. 7 and Fig. 8 indicates the magnitude of the velocity vector field, while its direction is represented by the direction vectors.

Furthermore, the uniformity index of velocity is computed on the outlet cross-sectional surface as defined in Eq. 7:

7 $$\gamma = 1 - \frac{1}{2}\frac{{{\text{ }}\int_{{{\text{outlet}}}} \left| {U - \bar{U}} \right|{\text{d}}{\text{A}}}}{{\bar{U}\int_{{{\text{outlet}}}} {\text{d}}{\text {A}}}}$$

where $U$ is the velocity magnitude and $\overline{U}$ refers to the surface-averaged velocity magnitude at the outlet cross section.

In accordance with Fig. 8, the uniformity index at the outlet cross section is 0.88 for HVAD and 0.76 for HM3.

### Hemocompatibility

In this section, the devices are compared in terms of hemocompatibility. The blood damage associated to each device is assessed by means of the estimated hemolysis index.

**Fig. 9:**
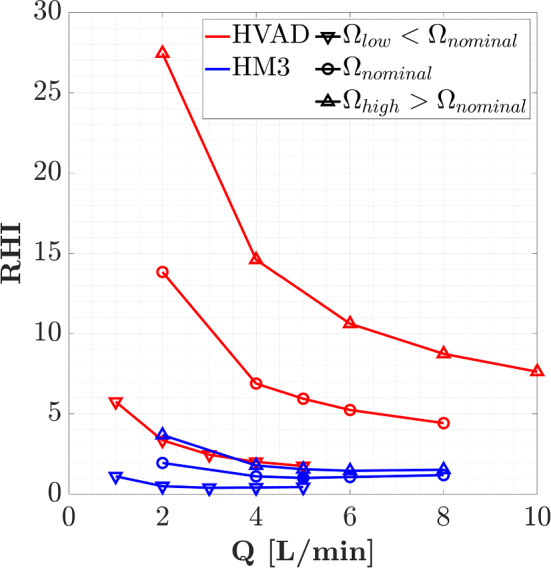
Relative hemolysis index against volumetric flow rate for HVAD and HM3 operating at different rotational speeds

Figure 9 shows the relative hemolysis index against the volumetric flow rate for both devices operating at different rotational speeds. The nominal rotational speeds are $\Omega_{{{\text{nominal}}}}^{{{\text{HVAD}}}} = 3000{\text{ rpm}}$ and $\Omega_{{{\text{nominal}}}}^{{{\text{HM3}}}} = 6000{\text{ rpm}}$, as defined above. Rotational speeds lower and higher than nominal ones are selected: $\Omega_{{{\text{low}}}}^{{{\text{HVAD}}}} = 1800{\text{ rpm}}$, $\Omega_{{{\text{low}}}}^{{{\text{HM3}}}} = 4000{\text{ rpm}}$, $\Omega_{{{\text{high}}}}^{{{\text{HVAD}}}} = 4000{\text{ rpm}}$, and $\Omega_{{{\text{high}}}}^{{{\text{HM3}}}} = 7000{\text{ rpm}}$.

**Fig. 10:**
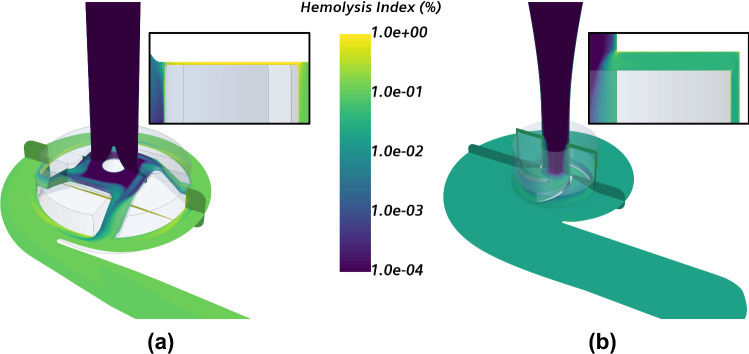
Hemolysis index field within **a** HVAD and **b** HM3, operating at nominal conditions (*Q* = 5 L/min, *∆p*_*t*_ ≅ 90 mmHg)

In addition, the hemolysis index field within both devices operating at nominal conditions is presented in Fig. 10, including a close view of the gap regions.

## Discussion

### Hemodynamic performance

As observed in Fig. 4 and Fig. 5, the pressure curves of HM3 have steeper slopes, which means that a change in the mean arterial pressure of the patient (as a consequence of a change in vascular resistance) will lead to less variations in flow rate, in comparison with HVAD which presents flatter pressure curves (Boës et al. 2019).

A significant difference in the maximum values of efficiency is detected in Fig. 5. Operating at nominal conditions, efficiencies of 47% and 27% are found for HM3 and HVAD, respectively. Moreover, operating at nominal speed, the maximum efficiency is achieved at a flow rate slightly higher than normal CO in HVAD ($Q = 6 {\text{L}}/{\text{min}}$), while the flow rate of maximum efficiency corresponds to $Q = 5 {\text{L}}/{\text{min}}$ in HM3.

Presumably, the decreased efficiency of HVAD owes to its design, namely the wide-blade impeller and the narrow gap, both required to produce hydrodynamic lift (Gil et al. 2022). The shape of its wide-blade impeller induces large areas of recirculating flow within the blade-to-blade passages leading to energy losses and, thus, to a decrease in efficiency. The extremely narrow gap, for its part, promotes elevated levels of shear stress. Moreover, the gap region is in contact with large blade tip areas and, therefore, elevated shear torque acts over a large surface of the impeller contributing to the increase in mechanical shaft power needed to rotate the impeller (Gil et al. 2022).

In the previous work by Gil et al. (2022), a non-conventional increment of efficiency was detected when increasing the gap clearance in HVAD, as a consequence of its gap design. Nevertheless, this tendency was only observed for gap clearances of the order of 40 μm, whereas the typical tendency (a decrease in efficiency when increasing the gap clearance) was detected for larger gap clearances, of the order of those found in HM3 (500–1000 μm), due to tip leakage. In this work, the tip leakage is quantified in percentage of the flow rate for both devices operating at nominal conditions. A 0.04% leakage flow is measured through the top gap of HVAD, whereas a 9% of the inlet flow is detected through the larger gaps of HM3. Thus, the narrow gaps in HVAD lead to considerably lower leakage losses, which means that the energy is incremented effectively in a larger portion of the inlet flow. Nevertheless, the windage losses in HVAD are expected to be significantly larger than in HM3, due to the elevated (shear) torque exerted by the blood over the impeller surfaces in contact with the gap region in HVAD. The windage loss is quantified as the amount of torque exerted on the impeller walls in contact with gaps in percentage of the total torque acting on the impeller of each device operating at nominal conditions. A 23% windage loss is found in HVAD, in contrast to a 9% windage loss in HM3. Therefore, the efficiency decline in HVAD is mostly caused by the (shear) torque production within the extremely narrow gap.

Furthermore, the torque (consisting of pressure and shear torque) exerted by the fluid over the impeller of HVAD is found to be increased by the shear component, especially on walls in contact with gaps. In the previous work by Gil et al. (2022), the shear torque was found to be of the same order of magnitude than the pressure torque in HVAD, whereas normally it is at least one order of magnitude lower, as occurs in HM3. This notably high shear torque in HVAD is a consequence of the high shear stress production within its gaps and contributes to the decline of efficiency in this device. The volumetric distribution of scalar shear stress shown in Fig. 6 demonstrates that a higher level of shear stresses is detected in HVAD compared to HM3, since larger volume fractions are subjected to high levels of stress. Moreover, slightly larger volumes are exposed to $\tau > 500~{\text{Pa}}$ than to $\tau \in \left[ {400,500} \right] {\text{Pa}}$ in HVAD, while the exposed volume decreases monotonously for increasing stress levels in HM3. Additionally, the volumetric distribution of stresses within HVAD follows a similar trend than that presented by Thamsen et al. (2015).

Regarding the fluid patterns presented in Fig. 7, significant zones of flow recirculation are detected within the blade passages of HVAD, due to its non-conventional shape. Smaller recirculation regions are detected in the external walls of the impeller. Although it incorporates conventional blades, i.e., similar to those found in typical turbopumps, HM3 also presents small zones of flow recirculation in the blade passages. Larger recirculation zones are detected in the 3D view of HM3 as well. Furthermore, the flow at the outlet cross section of HM3 is less uniform than that of HVAD, as detected in Fig. 8 and quantified through the uniformity index which is found to be 15% higher in HVAD. This is a consequence of the shape of the outlet cannula and diffuser in HM3 which are not aligned, leading to a large region of detached flow. Therefore, a further enhancement of performance in HM3 can be achieved with an optimized design that minimizes those recirculating and detached flow features.

### Hemocompatibility

For each device individually, higher hemolysis index is detected in Fig. 9 when increasing the rotational speed. Furthermore, a potentially higher risk of hemolysis is detected operating at low flow conditions, as encountered by other authors (Granegger et al. 2020; Thamsen et al. 2020). Operating at higher rotational speed the shear stresses are greater promoting higher levels of hemolysis, while the increase in hemolysis at low flow conditions is a consequence of the larger residence times of a blood cell within the pumps. For both devices operating at their nominal rotational speed, the hemolysis index is increased by a factor of around 2 at low flow rate ($Q = 2{\text{ L}}/{\text{min}}$) in comparison with the nominal flow rate condition ($Q = 5{\text{ L}}/{\text{min}}$). The increase in hemolysis, for both increasing speed and decreasing flow rate, is more pronounced for HVAD than for HM3. Moreover, the hemolysis index of HVAD operating at nominal conditions is six times greater than that obtained for HM3. This notably higher risk of hemolysis detected for HVAD is also associated to its non-conventional design. The extremely narrow gap clearances raise the magnitude of shear stresses within the gap region that extends over a large blade tip area, leading to an increase in hemolysis production.

Different trends are detected between the devices in Fig. 9. The hemolysis index decreases monotonously with flow rate in HVAD leading to an asymptotic tendency at high flow conditions, whereas a point of minimum hemolysis is found for HM3 corresponding to the operating conditions of maximum efficiency. This can be explained based on the scalar shear stress distributions presented in Fig. 6. The effect of flow rate over the distribution of stresses is negligible in HVAD, while an increase in the volume exposed to extremely high scalar shear stress is detected in HM3 at high flow rates. Therefore, in HM3 the high risk of hemolysis at high flow conditions owes to the larger volumes exposed to elevated levels of stress, whereas the high risk of hemolysis at low flow conditions is due to the larger residence times within the pump as explained above. On the contrary, the potentially higher risk of hemolysis in HVAD is dominated by the more elevated levels of stress promoted by its narrow gaps independently of the flow rate through the pump, as observed in Fig. 6 for $\tau > 300 {\text{Pa}}$.

Additionally, in Fig. 10 the main regions contributing to blood damage are detected. Gap regions and recirculation zones within the blade-to-blade passages are found to be the areas generating most part of the damage, especially for HVAD, since it presents larger areas of flow recirculation and gap clearances two orders of magnitude narrower than those found in HM3. Nevertheless, it can be seen how the blood downstream of the gap in HVAD has a lower level of hemolysis than the blood within the gap, as a result of the mixing of primary flow (99.96% of total flow, moderate level of hemolysis ~ 0.1%) and secondary flow or tip leakage (0.04% of total flow, high level of hemolysis ~ 1%).

Operating at nominal conditions, the maximum residence time of a blood cell is found to be 0.16 s and 0.08 s in the proximity of the impeller of HVAD and HM3, respectively. Moreover, the scalar shear stress reaches maximum values of 2000 Pa and 500 Pa within gap regions in HVAD and HM3, respectively. The volumetric distribution of stresses shown in Fig. 6 also reveals that HVAD presents notably larger volume fractions exposed to high levels of scalar shear stress than HM3: The percentage of volume subjected to $\tau > 500 {\text{Pa}}$—operating at nominal flow rate ($Q = 5{\text{ L}}/{\text{min}}$)—is 0.1% in HVAD and 0.0001% in HM3. Therefore, a globally lower hemolysis index field is expected for HM3, as observed in Fig. 10 and in consonance with previous Fig. 9.

## Conclusions

This work performed a comparison between the two main centrifugal LVADs of the latest generation—HVAD and HM3—considering both hemodynamics and hemocompatibility. An experimental validation of the CFD model was carried out as well.

The experimental validation showed a fair agreement between ex vivo and in silico results for both HM3 and HVAD in terms of pressure head.

The comparison between HVAD and HM3 revealed enhanced performance and hemocompatibility for HM3, since it presented higher hydraulic efficiency and lower levels of hemolysis. Operating at nominal conditions, the efficiency of HM3 (47%) was 70% greater than that of HVAD (27%), and the former had associated a hemolysis index six times lower than the latter. Moreover, the steeper pressure curves of HM3 are preferable to reduce the flow rate variations caused by changes in vascular resistance of the patient.

The non-conventional design of HVAD, involving a wide-blade impeller and narrow gaps, was found to be responsible for both the poor hemodynamic performance and the increased risk of blood damage compared to HM3. Moreover, HM3 was found to present a more appropriate performance than HVAD but could be further optimized to enhance efficiency and reduce blood damage. This implies that, despite the rapid development of these devices during the last decades, the more recent designs still need optimization. An important implication of this work is that more insight into the fluid dynamic performance of LVADs must be done owing to its direct relation with hemocompatibility. While most publications are focused on the evaluation of blood damage, an optimization of LVADs—from a turbomachinery point of view—is needed.

Furthermore, numerous operating conditions were considered in this work, demonstrating their influence over efficiency and hemolysis risk. An optimum point of maximum efficiency (and minimum hemolysis for HM3) was detected at each rotational speed. This implies that the clinician should adjust the rotational speed of the implanted LVAD in order to achieve an optimal flow rate through the pump, which, at the same time, have to be near the normal CO of the patient for normal values of mean arterial pressure.

The computational methodology followed in this work has some limitations. The main limitation is related to the experimental validation of the CFD model since no experimental tests have been conducted to measure shaft power and hemolysis index. Nevertheless, the obtained numerical results in terms of efficiency are in the range of those found in the literature concerning centrifugal LVADs, i.e. [20,50]% (Fraser et al. 2011; Wiegmann et al. 2018; Granegger et al. 2020; Hosseini and Keshmiri 2022), and the risk of hemolysis follows the expected tendency based on the literature as well (Fraser et al. 2012). In addition, two limitations must be mentioned regarding the numerical set-up of the model. Firstly, the blood is assumed to be a Newtonian fluid due to the high shear rates found in most of the domain; however, this assumption may be invalid in regions of the fluid with lower shear rates. Secondly, the hemolysis model assumes that blood is a continuum, but blood may behave as a non-continuum within the narrow gaps found in HVAD.

## Supplementary Information

Below is the link to the electronic supplementary material.

Supplementary file1 (DOCX 4659 KB)
