# Supplementary material for: Hemocompatibility and hemodynamic comparison of two centrifugal LVADs: HVAD and HeartMate3
Source: Biomech Model Mechanobiol. 2023 Jan 17;22(3):871–83. doi: 10.1007/s10237-022-01686-y (PMC10167126; doi:10.1007/s10237-022-01686-y)
Supplement: Supplementary file 1 — Supplementary file1 (DOCX 4659 KB) [file 10237_2022_1686_MOESM1_ESM.docx]

**Hemocompatibility and hemodynamic comparison of two centrifugal LVADs: HVAD and HeartMate3**

Antonio Gil^a^, Roberto Navarro^a^, Pedro Quintero^a^ and Andrea Mares^a^^[[1]](#footnote-1)^

a CMT-Motores Térmicos, Universitat Politècnica de València, Camino de Vera, s/n, Valencia, 46022, Spain

SUPPLEMENTARY MATERIAL

1. Mesh independence studies

A mesh independence study is performed for each device involving three grids. The mesh studies are carried out for several conditions operating at a nominal rotational speed. The results of these mesh studies in terms of efficiency and relative hemolysis index are represented in Figure 1 and Figure 2 respectively, and relative errors of performance variables for each device operating at nominal conditions are summarized in Table 1. In addition, Figure 3 shows the pressure distribution over the pressure and suction sides of blades in each device operating at nominal conditions, obtained with different mesh sizes. In this figure, the pressure coefficient, calculated as $c_{p}=\frac{p - p_{ref}}{\frac{1}{2}\rho\left( \Omega R_{imp} \right)^{2}}$, is represented along chord at the midspan of a blade. The x-axis coordinates are chosen to be the (non-dimensional) circumferential ($\theta^{*}$) and radial ($r^{*}$) coordinates for HVAD and HM3 respectively, owing to their different blade configuration and position, 0 corresponding to leading edge and 1 to trailing edge. As detected in those figures and table, the coarse meshes correctly capture the tendency of efficiency and hemolysis against flow rate (Figure 1 and Figure 2) and the distribution of pressure around blades (Figure 3), but these meshes lead to significant discrepancies in performance variables with respect to fine meshes (Table 1). Note that an elevated relative error in terms of hemolysis index is considered acceptable for HM3, due to its notably low absolute value (of the order of $0.01 \%$).

| Device | |  | HVAD |  |  | HM3 |  |
| --- | --- | --- | --- | --- | --- | --- | --- |
| Mesh | | Coarse | Medium | Fine | Coarse | Medium | Fine |
| Relative error | $\Delta p_{t}$ | $3.6 \%$ | $1.7 \%$ | $-$ | $0.5 \%$ | $0.4 \%$ | $-$ |
|  | $\eta$ | $8.4 \%$ | $3.7 \%$ | $-$ | $0.6 \%$ | $0.4 \%$ | $-$ |
|  | $HI$ | $5.5 \%$ | $1.3 \%$ | $-$ | $16.6 \%$ | $7.8 \%$ | $-$ |

Table 1 Results of the mesh independence studies, in terms of relative errors of performance variables at nominal operating conditions

Figure 1 Results of the mesh independence study: efficiency against volumetric flow rate, for (a) HVAD operating at *Ω_HVAD_* = 3000 rpm and (b) HM3 operating at *Ω_HM3_* = 6000 rpm

(a)


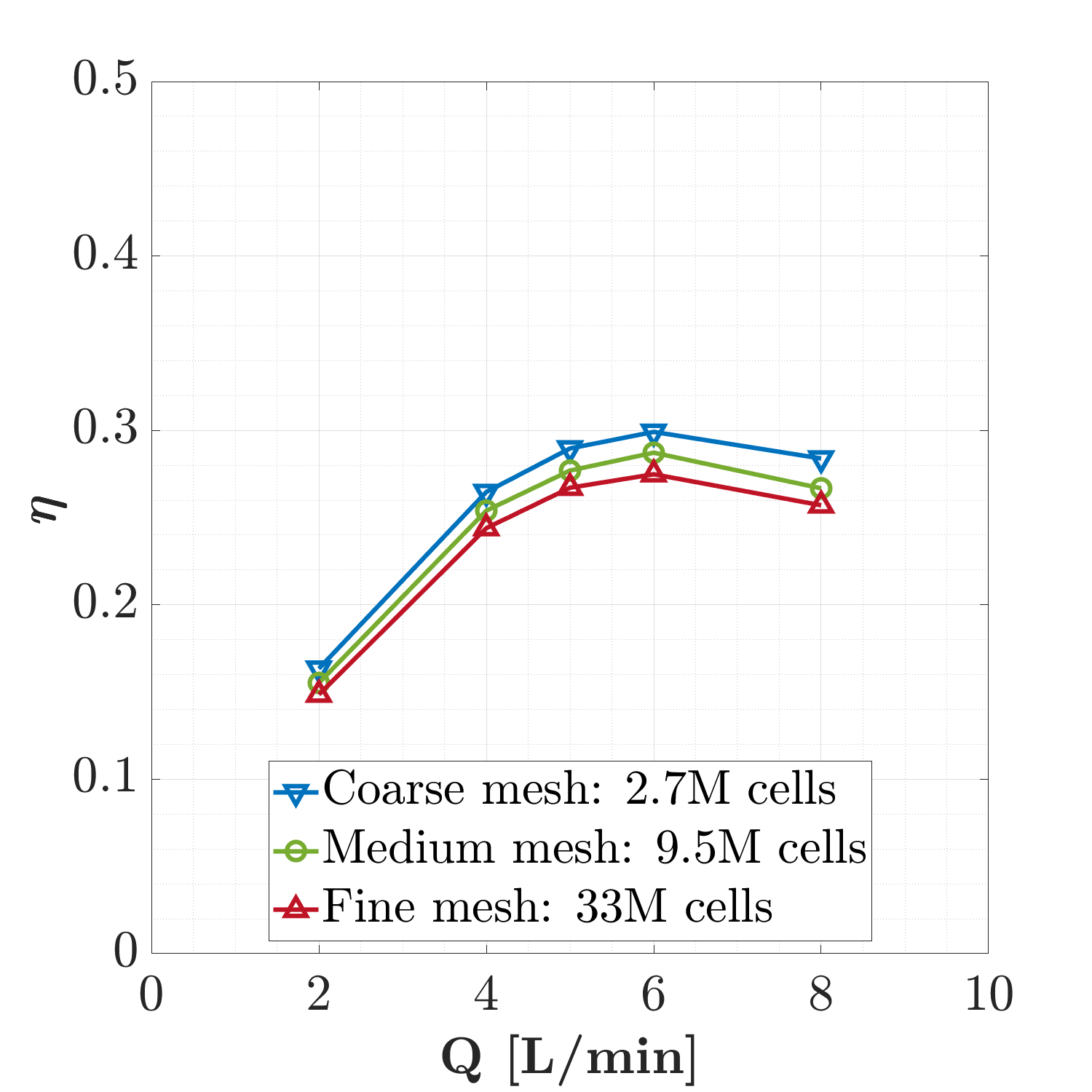


(b)


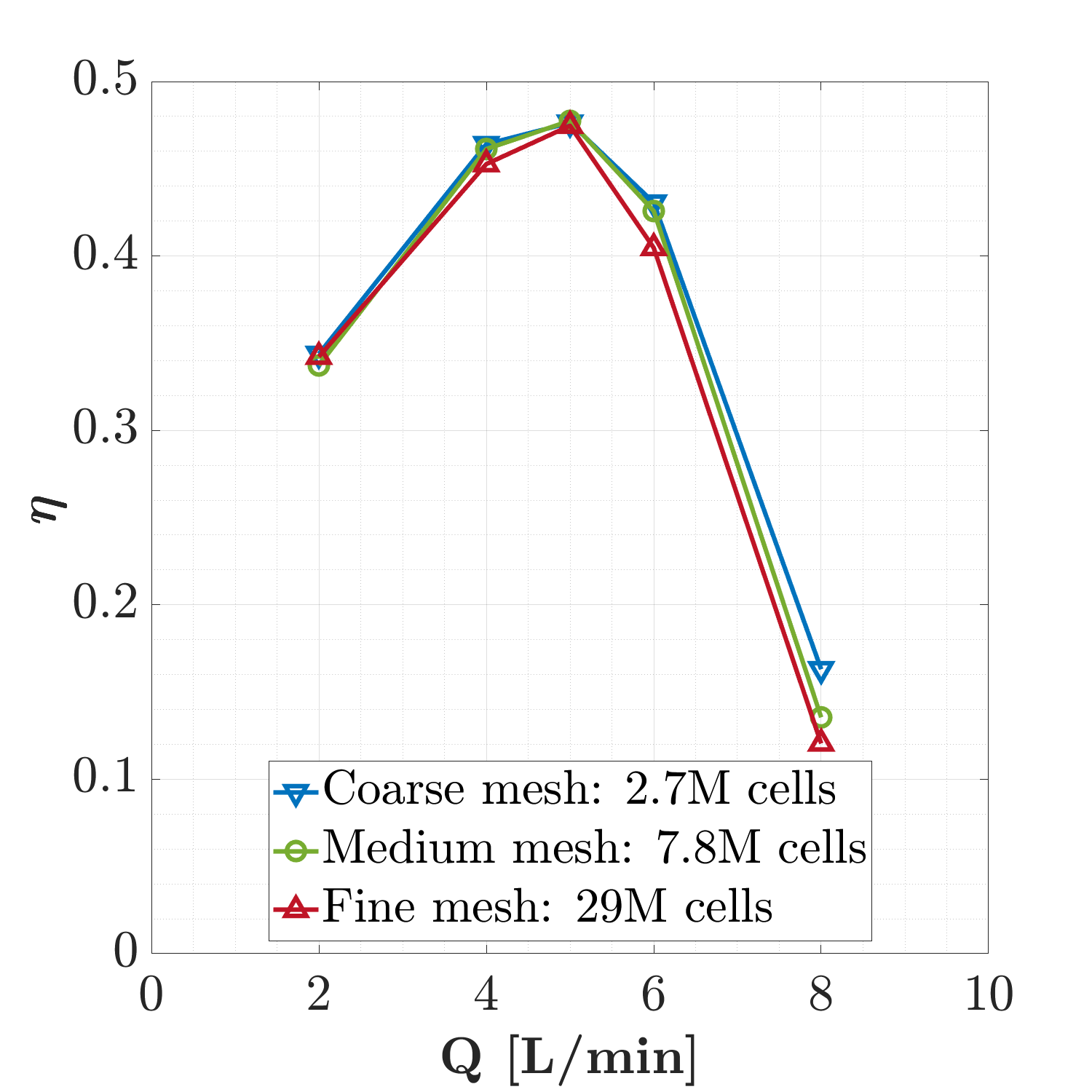


**Figure 2** Results of the mesh independence study: relative hemolysis index against volumetric flow rate, for **(a)** HVAD operating at *Ω_HVAD_* = 3000 rpm and **(b)** HM3 operating at *Ω_HM3_* = 6000 rpm, taking as reference value the hemolysis index obtained with the fine mesh for each device operating at *Q* = 5 L/min

(b)


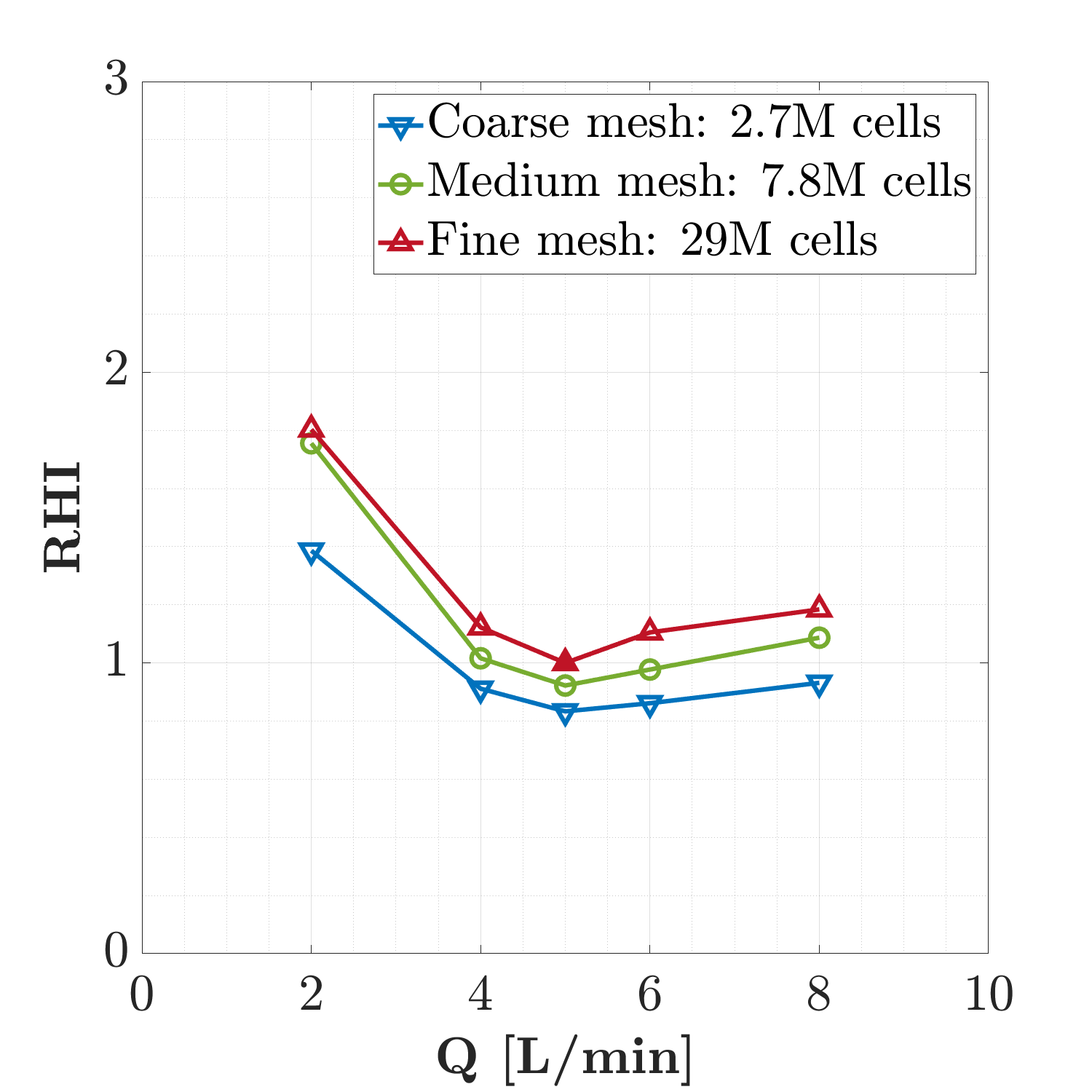


(a)


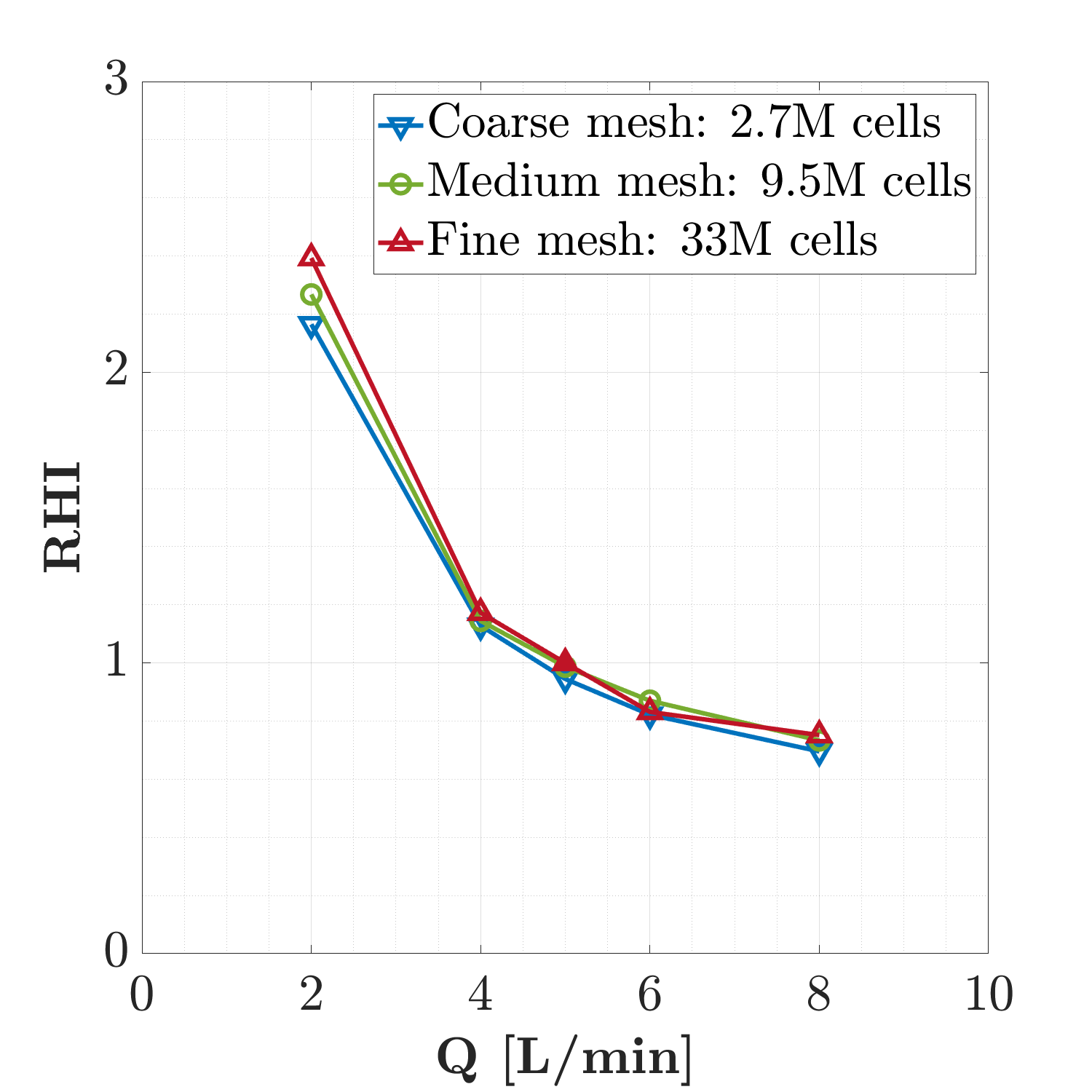


Figure 3 Pressure coefficient distribution along chord over the pressure and suction sides at the midspan of blades in (a) HVAD and (b) HM3, operating at nominal conditions (*Q* = 5 L/min, *∆p_t_* ≅ 90 mmHg), for several mesh sizes

(a)


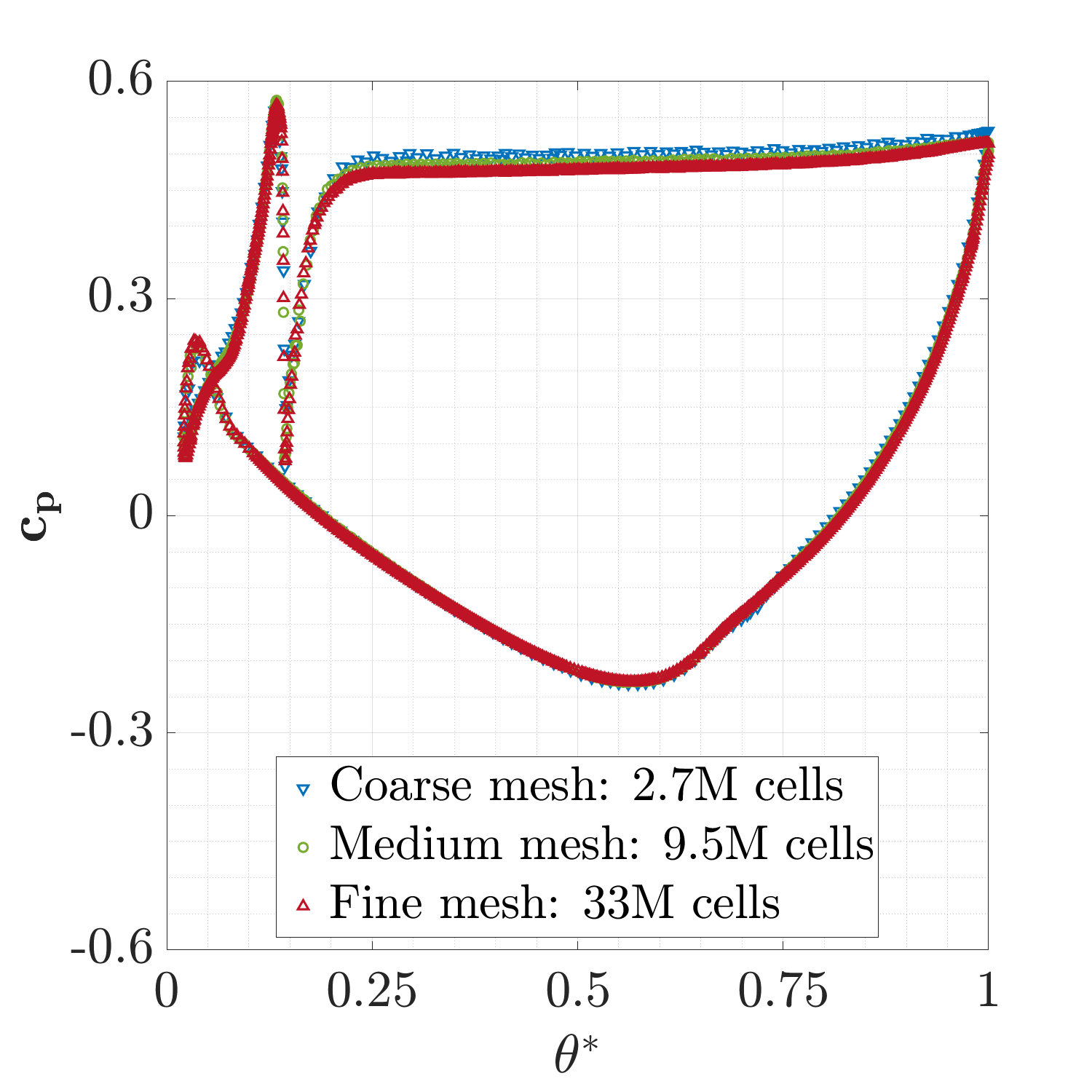


(b)


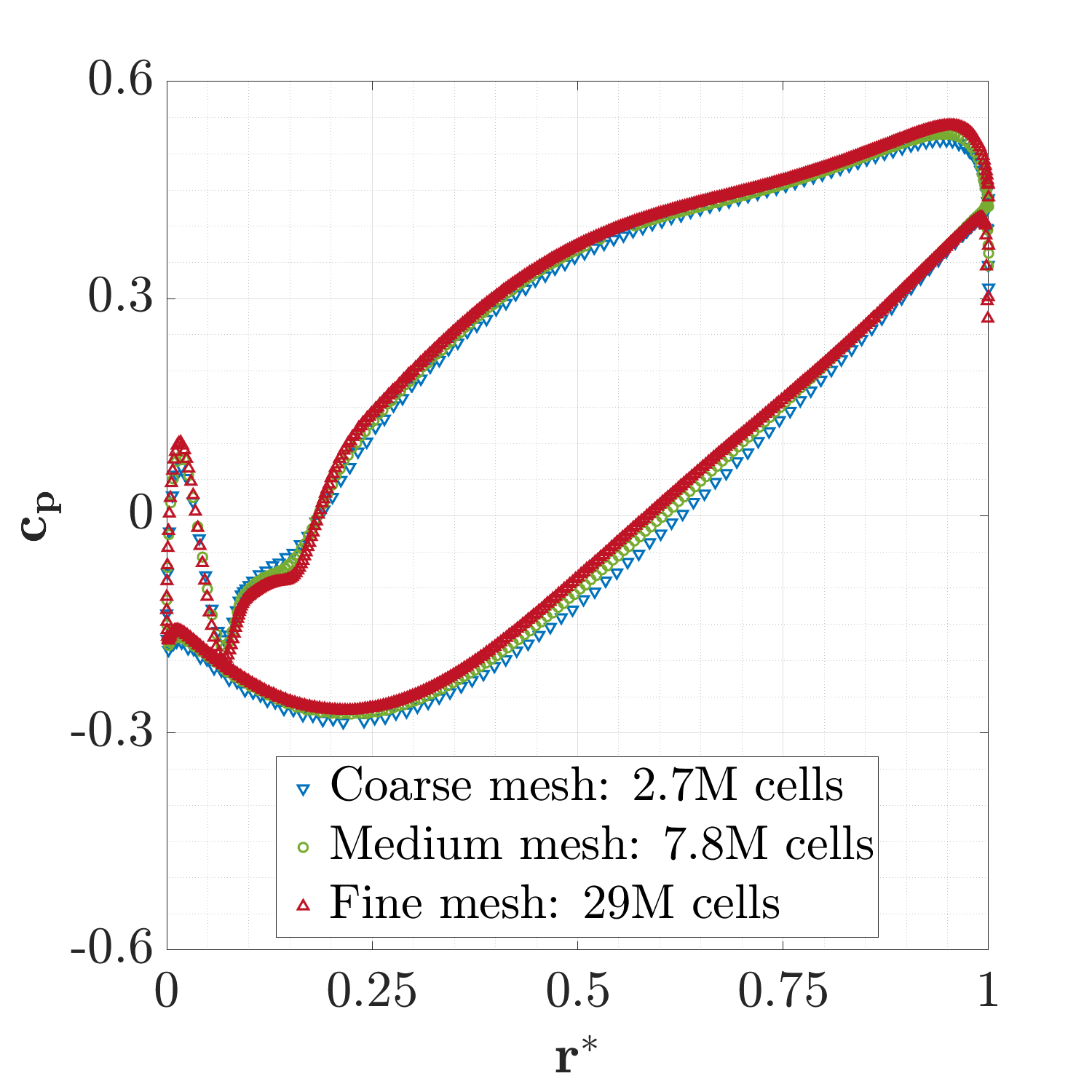


Pressure side

Suction side


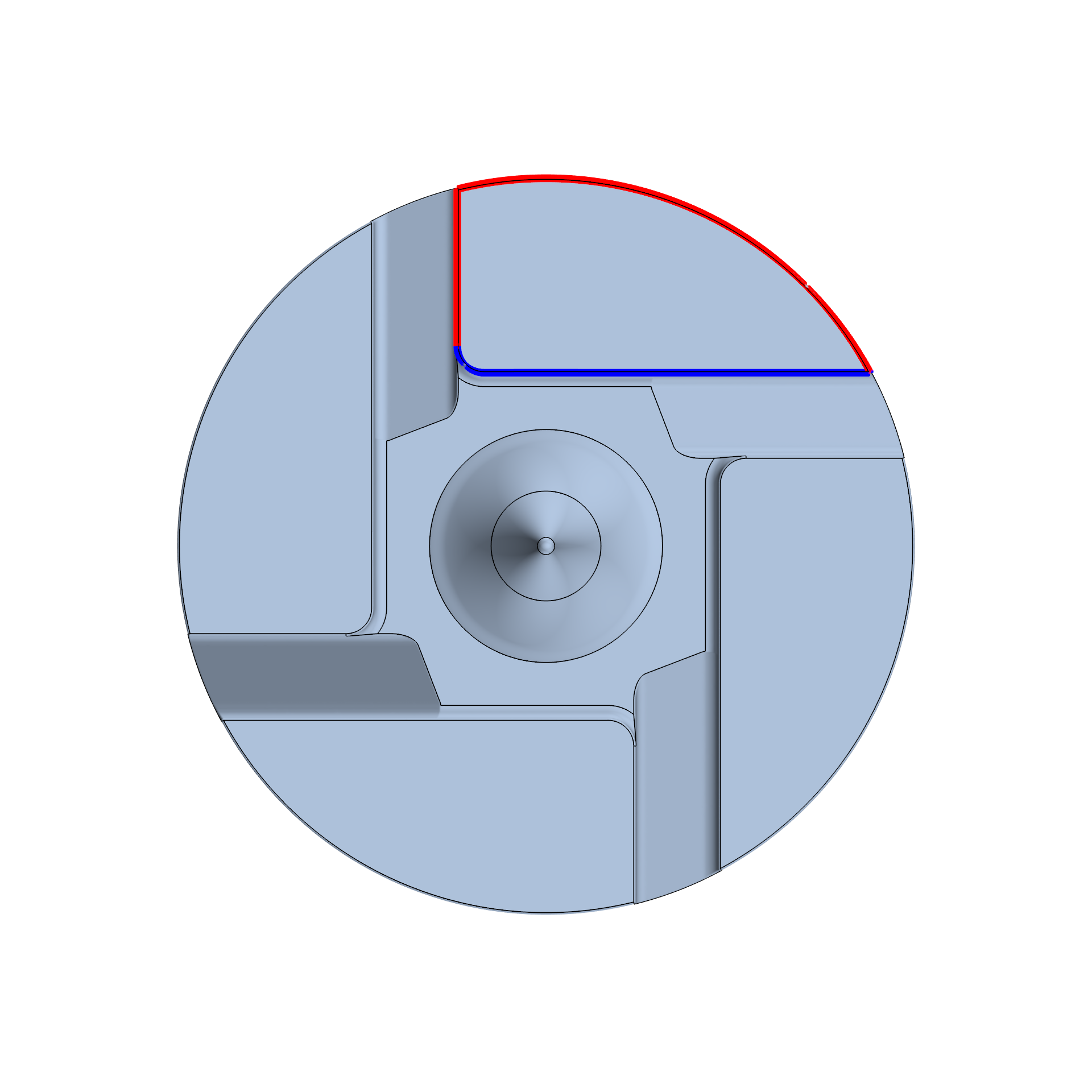

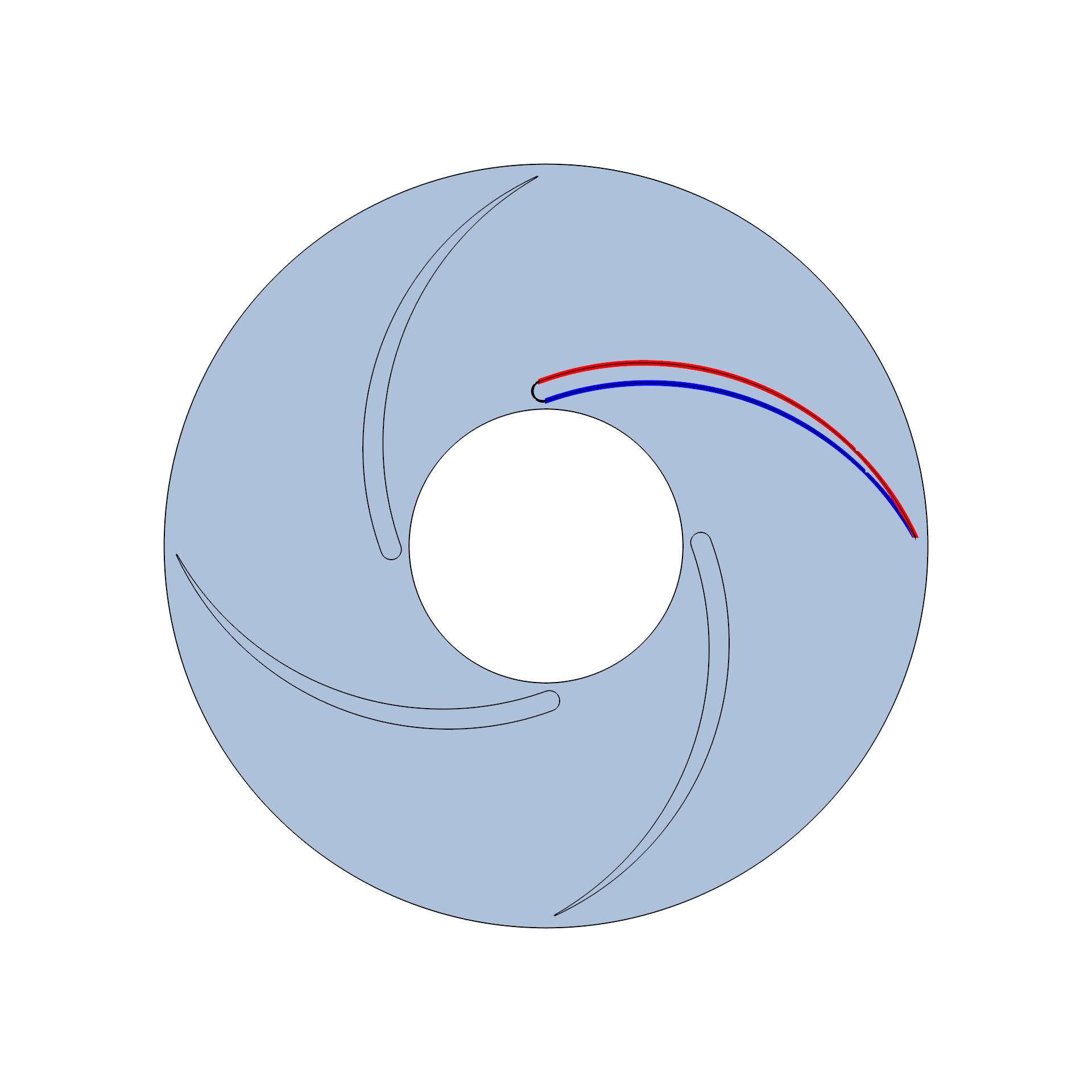


1. Comparison between steady- and unsteady-state results

A comparison between results obtained in steady- and unsteady-state simulations is performed in order to probe the quasi-stationary flow assumption and, thus, validate the MRF steady approach.

Figure 4 presents the pressure head curves, showing non-negligible discrepancies between RANS and (time-averaged) U-RANS results for HVAD. The mean and maximum relative errors in pressure head are 5% and 13% for HVAD and 3% and 7% for HM3.


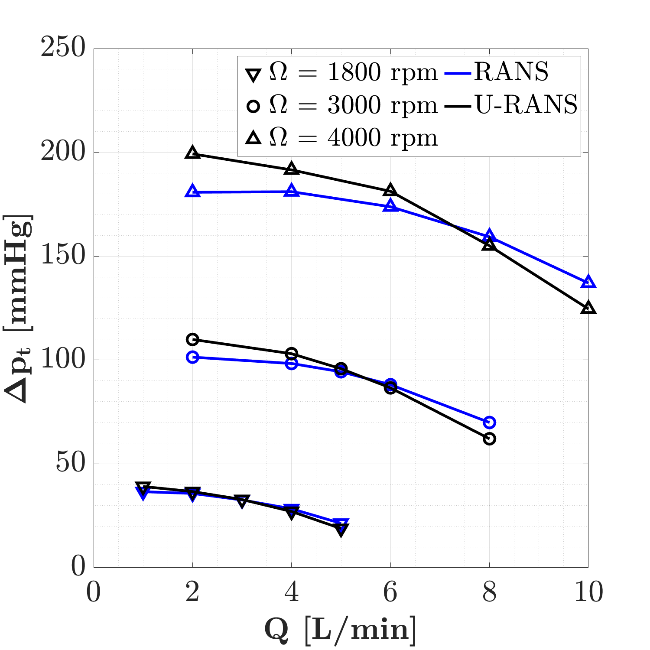


**(a)**


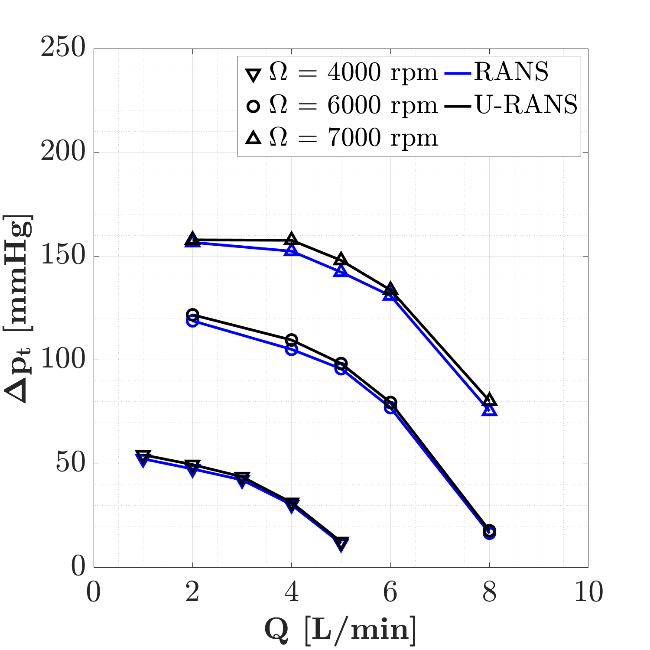


**(b)**

Figure 4 Pressure head curves against flow rate for several values of rotational speed, obtained in steady- and unsteady-state simulations: (a) HVAD and (b) HM3

Efficiency curves are represented in Figure 5, where the cumulative error from pressure head and power is manifested between RANS and (time-averaged) U-RANS results. This error is larger for HVAD, in consonance with previous Figure 4, while it is negligible at almost every operating condition for HM3. The mean and maximum relative errors in efficiency are 10% and 17% for HVAD and 2% and 11% for HM3.

The standard deviation of the transient evolution of each performance variable is calculated as well. For HVAD operating at nominal conditions, the standard deviations of pressure head and efficiency are 3.1% and 5.1% respectively. For HM3 operating at nominal conditions, in contrast, the standard deviations of pressure head and efficiency are 0.9% and 1.6% respectively.

**(a)**


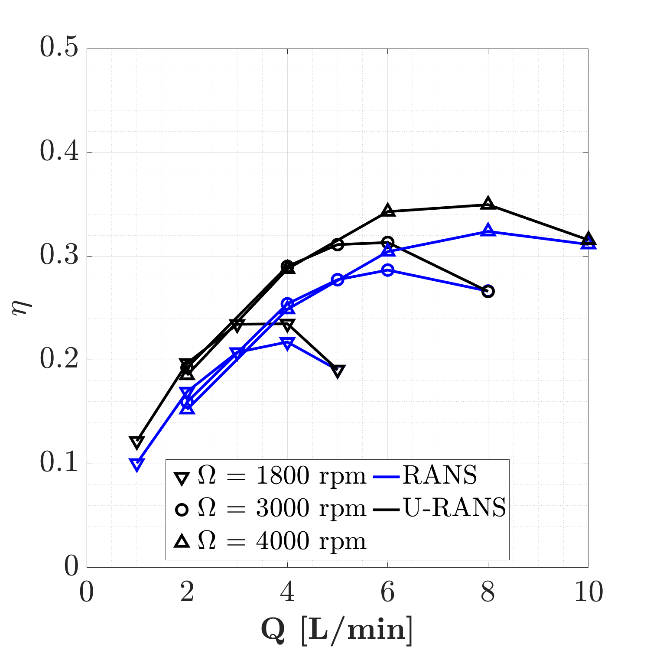

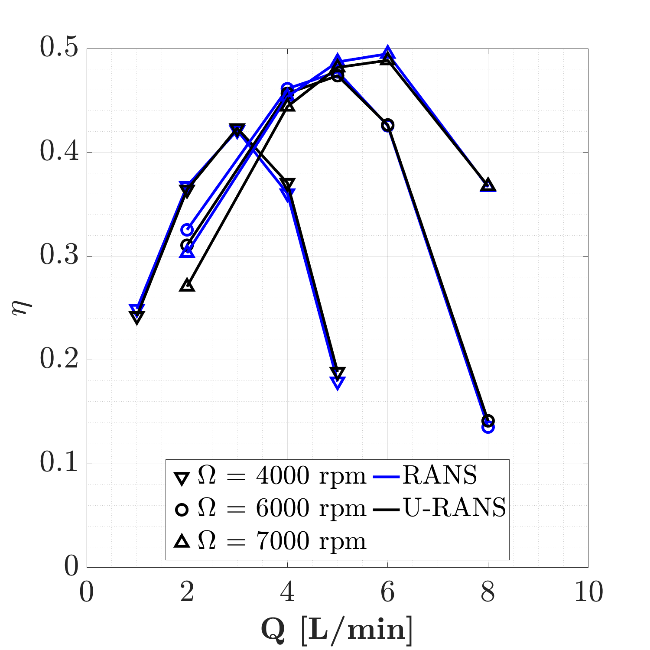


**(b)**

Figure 5 Efficiency curves against flow rate for several values of rotational speed, obtained in steady- and unsteady-state simulations: (a) HVAD and (b) HM3

The larger transient effects detected for HVAD are thought to be a consequence of its non-conventional design involving wide blades, since the shape of the wide-blade impeller promotes vortex shedding within the blade-to-blade passages, leading to high-amplitude fluctuations of performance variables.

Finally, the results of relative hemolysis index obtained in RANS and U-RANS simulations are compared in Figure 6. Again, larger discrepancies are found for HVAD as compared to HM3. The mean and maximum relative errors in hemolysis index are 15% and 20% for HVAD and 3% and 8% for HM3.


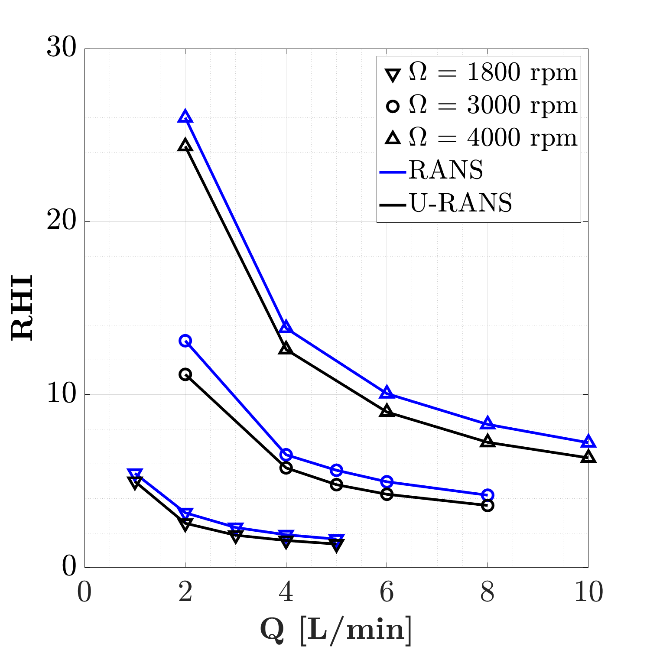


**(a)**


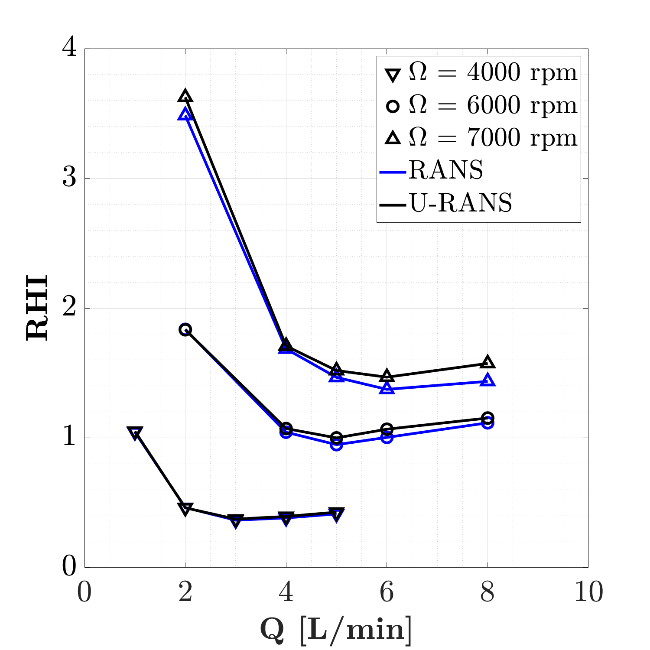


**(b)**

Figure 6 Relative hemolysis index curves against flow rate for several values of rotational speed, obtained in steady- and unsteady-state simulations: (a) HVAD and (b) HM3

Despite discrepancies found for HVAD operating at extreme flow conditions, the relative errors between RANS and time-averaged U-RANS results are found to be bounded, and the assumption of quasi-stationary flow can be applied at design and near-design conditions. Furthermore, the relative errors between steady and transient results are found to be negligible for HM3 and, thus, the hypothesis of quasi-stationary flow is assumed to be valid for HM3 through its entire operating map. Therefore, the MRF steady approach is assumed to be sufficiently accurate for characterizing the pumps.

1. 1 Corresponding author: [anmabo4@etsid.upv.es](mailto:anmabo4@etsid.upv.es) (Mares, Andrea). ORCID: 0000-0002-3720-6710 [↑](#footnote-ref-1)
